# Simple and Easily Interpretable Flap Monitoring Method Using a Commercial Pulse Oximeter and a Widely Used Bedside Patient Monitor

**DOI:** 10.7759/cureus.32549

**Published:** 2022-12-15

**Authors:** Daisuke Atomura, Sayaka Hoshino, Takeo Osaki, Shunsuke Sakakibara

**Affiliations:** 1 Department of Plastic Surgery, Kobe University Hospital International Clinical Cancer Research Center, Kobe, JPN; 2 Department of Plastic Surgery, Kobe University Graduate School of Medicine, Kobe, JPN; 3 Department of Plastic Surgery, Hyogo Cancer Center, Akashi, JPN

**Keywords:** bedside patient monitor, flap disorders, flap monitoring, photoplethysmography, reflectance pulse oximeter

## Abstract

Background

Various methods for monitoring after free flap surgery have been reported in the literature. Among them, pulse oximetry shows a sensitive reaction to vascular issues, and it is easy to interpret visually. However, previous reports used special equipment that was less commonly used and difficult to generalize. In this study, we used a commercial pulse oximeter and a widely used bedside patient monitor to monitor transplanted free tissue and lower extremities of healthy subjects with impaired circulation.

Methods

A reflectance pulse oximeter sensor was attached on the flap after free tissue transplantation. The sensor was connected to a bedside patient monitor, and the flap oxygen saturation (SpO_2_) levels and arterial waveforms were continuously monitored. Additionally, blood circulation disorder was induced in the lower limbs of healthy volunteers using pressure cuff inflation on the thigh, and the waveform and SpO_2_ levels on the pulse oximeter attached to the lower leg were monitored.

Results

Twenty-two patients were included in this study. No postoperative vascular issues were observed in any case. Pulse oximeters showed normal rhythmic wavelengths, and the flap SpO_2_ level ranged approximately >90%. The pulse oximeter waveform rapidly disappeared during arterial occlusion in the thigh pressure cuff inflation test, and the waveform flattened and the SpO_2_ level decreased slightly during venous congestion.

Conclusion

Flap monitoring using a commercially available pulse oximeter and a bedside patient monitor is a versatile, easy-to-interpret, and useful method. Changes in waveform and SpO_2_ levels appear during arterial and venous circulation disorders, and these changes can be differentiated.

## Introduction

In free flap reconstruction, occlusions of the anastomotic vessels are critical complications; therefore, postoperative monitoring is important for flap salvage. Various methods of flap monitoring have been reported in the literature of reconstructive surgery, including clinical observation, pinprick testing, blood glucose measurement [1], sound Doppler test [2], color duplex ultrasonography [3], microdialysis [4], implantable venous Doppler test [5], laser Doppler flowmetry [6], and near-infrared spectroscopy (NIRS) [7], and each method has its advantages and disadvantages.

The ideal monitoring method for free flaps should be harmless to both the patient and flap; moreover, it should be rapidly responsive, accurate, reliable, and applicable to all types of flaps. Furthermore, it should be equipped with a simple display so that even relatively inexperienced personnel can identify the development of circulatory impairments [8]. However, currently, no method satisfies all of these requirements, and there is no single reliable method for early detection of arterial or venous complications.

It has been reported that most of the thromboses occur within the first two postoperative days, and early detection of thrombosis is thought to increase the flap salvage rate [9]. Thus, continuous monitoring is an ideal method for early detection of obstruction. Among the abovementioned types of monitoring, an implantable venous Doppler test, laser Doppler flowmetry, and NIRS methods are considered as continuous methods; however, all the methods have the disadvantages of the need for special equipment, some skill in discriminating circulatory disorders, and high cost. Recently, there has also been interest in monitoring both labor and device costs [10]. With the advent of new-albeit usually more expensive devices, current research on valve monitoring has become more accurate.

As a simple and low-cost method for continuous monitoring, pulse oximetry based on photoplethysmography has been introduced, and its usefulness has been reported [11-15]. Pulse oximetry as a monitoring method has been demonstrated to provide a sensitive reaction to vascular complications, and it is easy to interpret visually. However, previous reports indicated the need to use a self-made device or software, which is not so common and might not be very versatile, to interpret the data.

In this study, we investigated a more economical and easy-to-interpret method called pulse oximetry-based monitoring using a commercial pulse oximeter and a widely used bedside monitor. Additionally, a model of circulation disorder in the lower leg perforator of healthy subjects was developed using a tourniquet and changes in pulse oximeter waveforms, and arterial hemoglobin oxygen saturation (SpO_2_) levels during arterial occlusion and venous congestion were measured.

## Materials and methods

Principles of pulse oximetry and MAX-FAST

There are two main principles of pulse oximetry. First, using the difference in absorption spectra between oxygenated and deoxygenated hemoglobin, the detector receives red and infrared light emitted from the emitter, and the ambient light is subtracted to analyze the measured value. Second, pulse oximetry is based on photoplethysmography, which is a simple and low-cost optical technique that can be used to detect blood volume changes in the microvascular bed of tissue [16]. Arterial and venous blood, tissue, and bone are present in the tissue, and these factors affect the absorptivity of red and infrared light. Among these factors, the part of the volume change that appears as a pulse wave is entirely due to arterial blood, and by extracting these data, the SpO_2_ levels can be measured accurately [17].

The skin reflectance pulse oximeter (Nellcor™ MAX-FAST™, Medtronic, Dublin, Ireland) is a type of pulse oximeter that is mainly attached to the patient’s forehead. This method is frequently used in the anesthesiology field, which requires general systemic management [18,19]. The MAX-FAST sensor is manufactured to target the supraorbital artery, and it measures arterial waves at a measurement depth of 2-3 mm [20]. It is compatible with the Philips monitoring platform IntelliVue MX700 (Philips, Eindhoven, the Netherlands), which is widely used as a bedside patient monitor; hence, by simply connecting it via a cable, the waveform can be easily visualized without a special monitoring system.

Monitoring and measurement in the transplanted free tissue

**Figure 1:**
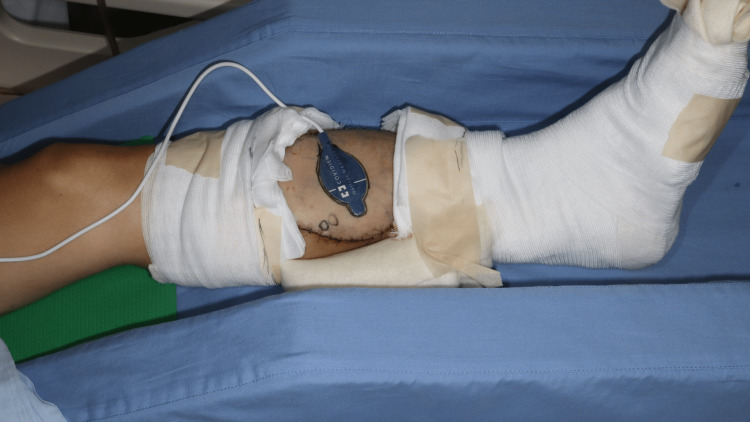
A case of ALT transplantation in the lower leg MAX-FAST is attached directly above the perforator in the flap. ALT: Anterolateral thigh Manufacturer details Nellcor™ MAX-FAST™: Medtronic, Dublin, Ireland

**Figure 2:**
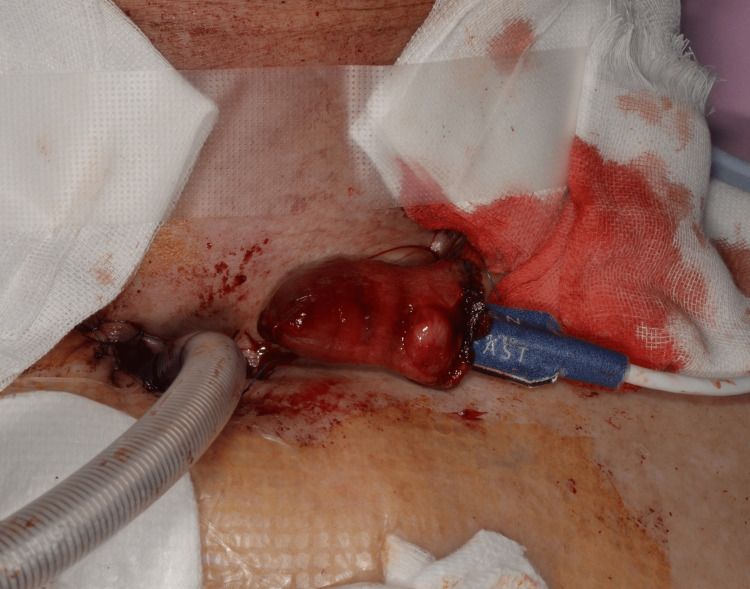
MAX-FAST attached to the free jejunal monitoring flap MAX-FAST was attached to the mucosal side of the exteriorized sentinel monitoring flap. Although mucus exudation was observed, MAX-FAST was well attached. Manufacturer details Nellcor™ MAX-FAST™: Medtronic, Dublin, Ireland

Among the free tissue transfers performed at the Hyogo Cancer Center (Akashi, Japan) from June 2017 to March 2022, we excluded cases of oral cancer reconstruction and buried flap as well as cases with small skin paddles that were unsuitable for sensor attachment from this study. The MAX-FAST sensor was used for flap monitoring. In cases of skin flaps, the sensor was attached in such a way that the midpoint between the light emitter and the detector was directly over the perforator of the flap, which can be detected using a sonic Doppler (Figure 1). In cases of free jejunum wherein the waveforms and values ​​measured from the buried jejunum seemed unreliable, the sensor was attached to the mucosal side of the exteriorised sentinel monitoring flap (Figure 2).

**Figure 3:**
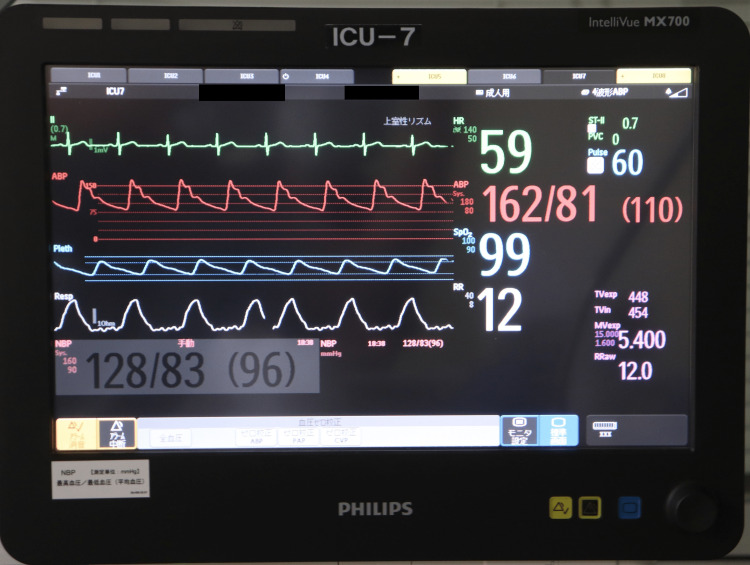
Flap arterial waveform and SpO2 levels projected on a bedside patient monitor (IntelliVue MX700) The blue graph in the center shows the arterial waveform of the flap. In this case, a continuous arterial waveform was shown, and SpO_2_ remained at the level of 95%–100%. Manufacturer details IntelliVue MX700: Philips, Eindhoven, the Netherlands

The sensor was connected to a bedside patient monitor (IntelliVue MX700, Philips, Eindhoven, the Netherlands) to continuously observe arterial waveforms and flap SpO_2_ levels (Figure 3).

Simultaneously, specialized nurses conducted a handheld Doppler examination of the arterial and venous pulses of the flap every two hours. Postoperative monitoring was started in the intensive care unit and continued up to three postoperative days. Saturation to monitor the patient’s general condition was displayed on another monitor with a small pulse oximeter device attached to the finger.

Pulse oximeter waveform and value measurement test during arterial occlusion and venous congestion

**Figure 4:**
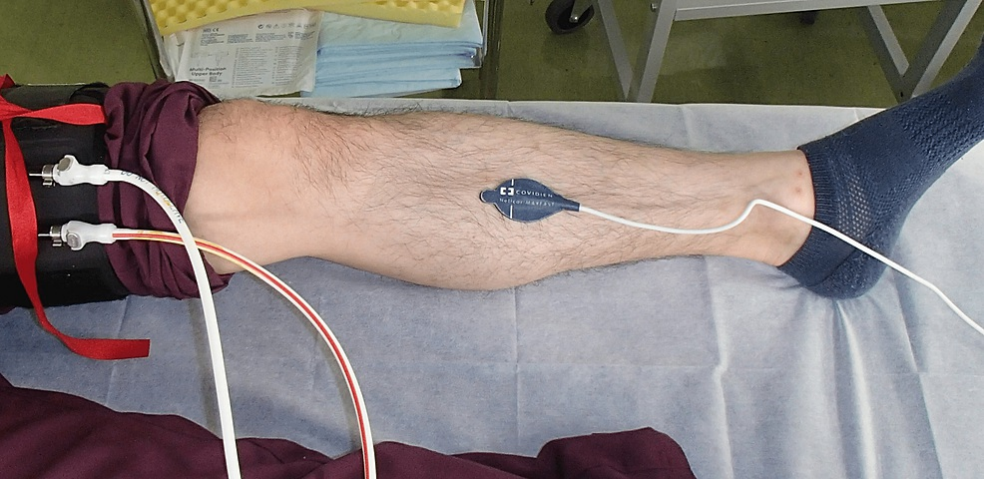
Lower-limb thigh pressure cuff inflation test in healthy subjects A tourniquet was attached to the thigh of the subject, and MAX-FAST was attached on the posterior tibial artery perforator of the lower leg. Changes in pulse oximeter waveform and SpO_2_ levels before and after cuff inflation were recorded. Manufacturer details Nellcor™ MAX-FAST™: Medtronic, Dublin, Ireland

By inducing blood circulation disorder in the lower limbs of the healthy subjects, changes in the pulse oximeter attached to the lower legs were observed. Four healthy adult volunteers were included in this study, and an arterial occlusion and a venous congestion model were developed using a tourniquet for seven lower limbs (one limb was excluded because of varicose veins). The posterior tibial artery perforator of the lower leg was confirmed using a handheld sound Doppler, and MAX-FAST was attached to the skin directly above it (Figure 4).

A tourniquet was attached to the ipsilateral thigh of the lower leg, and the cuff pressure was increased above systolic blood pressure for one minute as an arterial occlusion model and above diastolic blood pressure for five minutes as a venous congestion model, thereby causing blood circulation disorder in the lower leg. Thereafter, waveform changes and SpO_2_ levels before and after cuff inflation were observed, respectively. This study was approved by the institutional ethics committee of the Hyogo Cancer Center (R-904).

## Results

Monitoring and measurement in the transplanted free tissue

Overall, 22 patients who had undergone breast, facial, limb/trunk, and hypopharyngeal reconstructions with free jejunum were included in this study. No patient had a history of respiratory or heart disease that could affect general saturation. Table 1 summarizes the clinical data. Among all cases, there were no cases of vascular complications, return to the theater for re-exploration, or flap loss.

**Table 1:** Summary of the clinical cases DIEP: Deep inferior epigastric perforator flap; RF, radial forearm flap; RAM, rectus abdominis musculocutaneous flap; LD, latissimus dorsi flap; ALT, anterolateral thigh flap; FJ, free jejunum flap.

Case	Age (years)	Gender	Flap type	Recipient site	Vascular complications
1	68	Female	DIEP	Breast	No
2	57	Female	DIEP	Breast	No
3	56	Female	DIEP	Breast	No
4	58	Female	DIEP	Breast	No
5	54	Female	DIEP	Breast	No
6	47	Female	DIEP	Breast	No
7	59	Female	DIEP	Breast	No
8	45	Female	DIEP	Breast	No
9	46	Female	DIEP	Breast	No
10	53	Female	DIEP	Breast	No
11	81	Male	RF	Nose	No
12	55	Male	RAM	Maxilla	No
13	42	Male	RAM	Cheek	No
14	53	Male	LD	Abdominal wall	No
15	73	Male	ALT	Forearm	No
16	56	Male	ALT	Lower leg	No
17	78	Female	ALT	Axilla	No
18	68	Female	Scapular	Buttock	No
19	73	Female	LD	Buttock	No
20	65	Male	FJ	Hypopharynx	No
21	75	Male	FJ	Hypopharynx	No
22	52	Male	FJ	Hypopharynx	No

**Figure 5:**
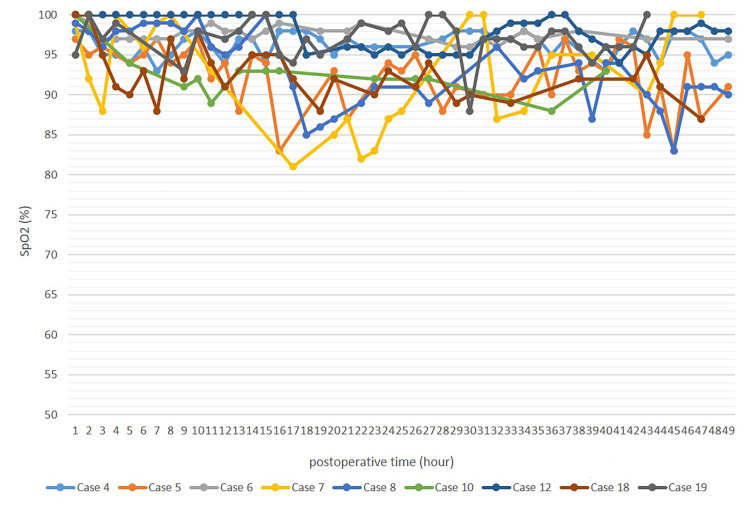
Course of the SpO2 level up to 48 hours after flap transfer The data of nine cases that could be extracted from the instrumental records are shown. The SpO_2_ level was generally >90%, although there were missing values and a temporary decrease due to body movements.

The pulse oximeter indicated normal and rhythmic wavelengths with no waveform disappearance or flattening. Although the flap SpO_2_ level sometimes showed a transient decrease, this was not persistent, ranging from approximately 90% to >90%. Figure 5 shows the course of nine cases wherein SpO_2_ levels could be extracted from the monitoring records for up to 48 hours postoperatively. There were some missing values ​​and a transient drop in the SpO_2_ level, which was mostly >90%, due to body movement.

Pulse oximeter waveform and value measurement test during arterial occlusion and venous congestion

**Figure 6:**
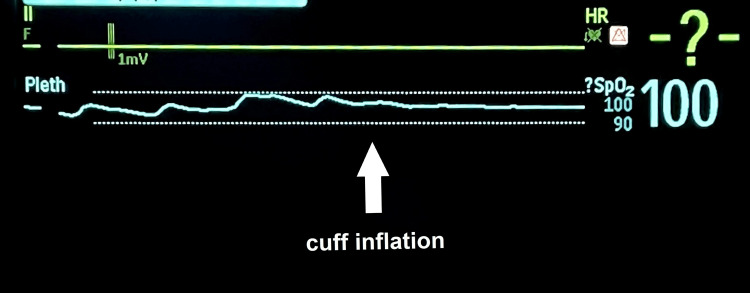
Waveform change of the pulse oximeter during arterial occlusion After cuff inflation (arrow) of the thigh over systolic, the pulse oximeter waveform on the lower leg was lost immediately.

Immediately after the cuff inflation of the thigh over the systolic pressure, the pulse oximeter waveform on the perforator of the lower leg disappeared rapidly, and the blood circulation disorder was rapidly monitored (Figure 6). Shortly after the disorder, the SpO_2_ level became unobservable.

**Figure 7:**
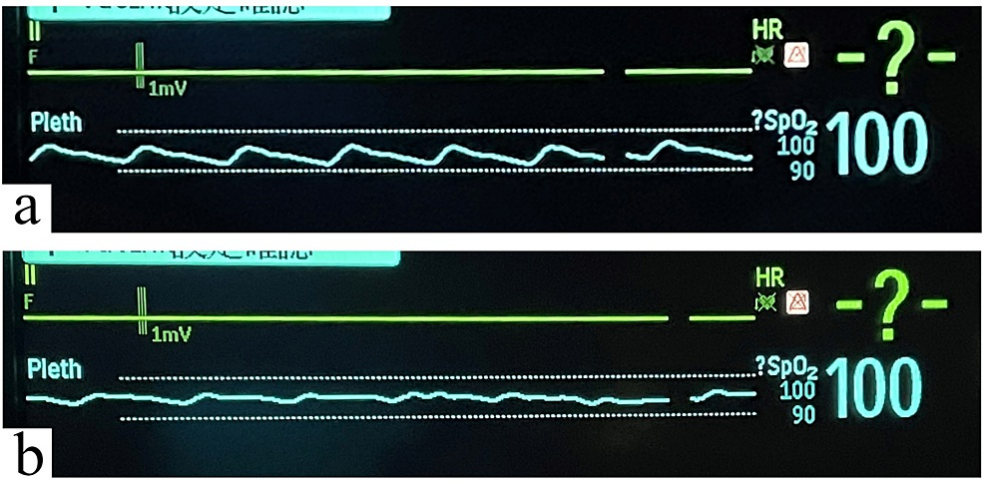
Waveform change of the pulse oximeter before and during venous congestion (a) Before venous congestion. A good waveform was projected to the monitor. (b) During venous congestion. Shortly after cuff inflation of the thigh over the diastolic pressure, the pulse oximeter waveform on the lower leg gradually flattened.

**Figure 8:**
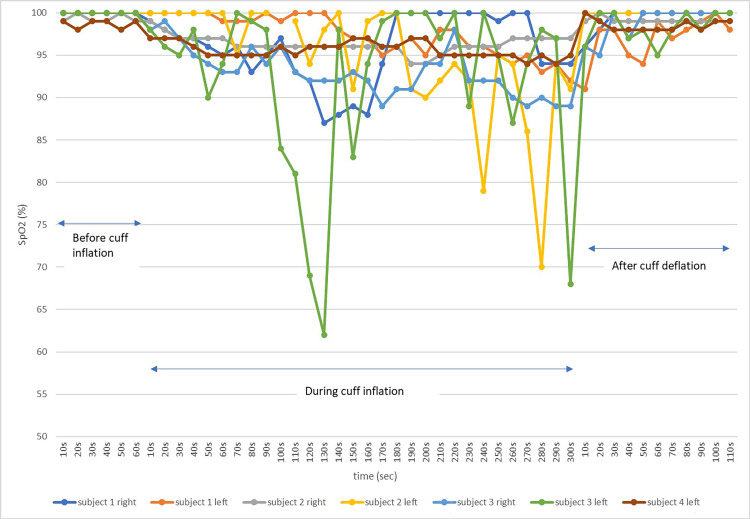
Changes in SpO2 levels before and after venous congestion in seven limbs of healthy subjects Before cuff inflation, the SpO_2_ level showed a high value with almost no fluctuations. After cuff inflation, the SpO_2_ level showed a slight decrease and often showed up-and-down fluctuations. After cuff deflation, the SpO_2_ level was improved to a high value.

Figure 7 shows the waveform change before and after cuff inflation of the thigh over the diastolic pressure. Soon after cuff inflation, the pulse oximeter waveform on the lower leg gradually flattened and the low perfusion alert sounded. Figure 8 shows the course of SpO_2_ levels before and after cuff inflation. Before cuff inflation, the SpO_2_ level showed almost no change and was approximately 100%. After cuff inflation, the SpO_2_ level showed a slight decrease of approximately 5%. During the five-minute cuff inflation, the SpO_2_ level remained generally low, often showing up-and-down fluctuations. SpO_2_ levels improved after cuff deflation, and the value was almost the same as before cuff inflation.

 

Representative patients

Case 1

A 56-year-old man had a solitary fibrous tumor on his left lower leg. The tumor was resected, including the deep fascia and tibial periosteum, and the defect was reconstructed using an anterolateral thigh (ALT) flap. Postoperatively, MAX-FAST was attached to the ALT flap (Figure 1), and the waveform and SpO_2_ were monitored using the IntelliVue MX700. Thereafter, the flap survived, the monitoring waveform reflected good arterial waveforms, and the SpO_2_ level remained at 95%-100%.

Case 2

A 75-year-old man with hypopharyngeal cancer who underwent pharyngolaryngectomy. After tumor resection, reconstruction of the circumferential pharyngeal defects was performed using a free jejunum flap. Postoperatively, MAX-FAST was attached to the mucosal side of the exteriorized sentinel monitoring flap (Figure 2), and the waveform and SpO_2_ level were monitored using the IntelliVue MX700. Although the flap survived, the waveform disappeared temporarily because of intestinal peristalsis, which mostly reflected good arterial waveforms and SpO_2_ levels.

## Discussion

The monitoring method of using a commercial pulse oximeter after free tissue transplantation was simple, noninvasive, and easy-to-interpret by nursing staff. Recently, flap checks by resident doctors have become less common because of time and labor constraints, and the need to rely on ancillary nonresident staff and new technology has been reported [21]. By implementing the use of a pulse oximeter that the nursing staff is accustomed to using, it is easy to judge the blood flow disorder of the flap; moreover, it is easy to share information when observing the flap condition. Unlike the probe type, the reflectance pulse oximeter is safe with less heat damage. Furthermore, the IntelliVue monitor compatible with MAX-FAST is widely used at clinical sites, and we believe that this monitoring method is easy to apply clinically.

There have been several reports on monitoring methods using pulse oximetry. Kyriacou et al. monitored DIEP flaps using a custom-made three-wavelength reflective photoplethysmography sensor and the system development software LabVIEW [15]. They concluded that this method was useful for achieving the viability of free flaps, and it successfully evaluated the viability. However, its use is not very common in clinical practice, and it is difficult for staff to understand.

NIRS, laser Doppler flowmetry, and implantable venous Doppler are often reported as continuous monitoring methods. Smit et al. reported that NIRS has the most potential in becoming the ideal monitoring method [9]. NIRS is a noninvasive and simple method with a high positive and negative predictive value. However, as the precise cutoff value of the circulation disorder in actual clinical settings has not been established and because the numerical trends must be evaluated over time [7], it is somewhat less objective in terms of discriminating circulation disorder and takes time to determine anastomotic embolism. In addition, although tissue oxygen saturation becomes stable after 12 hours of operation, it is unstable in the early stage after the operation, and it might be misunderstood as a vascular complication [22]. Similar to NIRS, laser Doppler flowmetry has an unclear cutoff value, and it is necessary to evaluate circulation disorders based on the trend [6]. The implantable venous Doppler method is a direct and fairly reliable blood flow evaluation technique, but it is an invasive method and there are concerns about infections.

Recently, the costs of monitoring have raised concerns [10,23]. When the monitoring method uses a newly developed device, the equipment itself becomes more expensive as the accuracy increases, and the overall cost increases accordingly. All three abovementioned methods have the disadvantages of requiring special equipment and high cost, and the costs of the monitoring box and disposable probe or sensor are $3100 and $412, $5,460 and $101, and $16,500 and $150, respectively [9]. The cost of MAX-FAST used in this study was low, i.e., approximately $94 each [20]. Regarding the monitoring box, we used a bedside monitor from Philips that is very commonly used in clinical practice; thus, no special equipment was required.

Photoplethysmography detects changes in vascular volume synchronously with the heart rate, and it has been theorized that the waveform signal sharply and accurately disappears during arterial occlusion. However, it is unclear what changes would occur during venous congestion. Lindsey et al. reported that the waveform on the pulse oximeter was lost during arterial obstruction, and the waveform was flattened during venous obstruction [12]. Agashe et al. reported that SpO_2_ levels decreased within a few minutes when MAX-FAST was attached to the forehead and the measurement site was congested as a Trendelenburg position, and it recovered when the congestion was released [24]. In this study, using an arterial occlusion and venous congestion model, we measured how the waveform and pulse oximeter readings changed, and the results were similar to previous reports as follows: the pulse oximeter waveform disappeared rapidly during arterial occlusion, the waveform flattened and SpO_2_ levels decreased during venous congestion. In cases of arterial occlusion, it can be easily concluded that the waveform disappears with the arterial pulsation. During venous congestion, increased venous resistance made arterial inflow difficult, and the waveform was considered to be flattened. It is considered that as the SpO_2_ level is measured by extracting only the arterial pulsation component, the SpO_2_ level does not decrease during venous congestion; however, there is a slight change in venous volume, and the venous pulsation artifact is accidentally measured, resulting in a decrease in SpO_2_ levels.

The main limitation of our study is that changes in the waveform signal and SpO_2_ level were uncertain when anastomotic thrombus actually occurred; moreover, there are no cases of anastomotic thrombus in both arteries and veins in clinical cases, and the cutoff value could not be clarified. However, the cuff inflation test in healthy subjects showed that some changes in the waveform and SpO_2_ levels appeared during blood circulation disorders. In particular, the changes were quite sensitive and obvious to everyone during arterial occlusion. In actual clinical congestion, venous return is almost completely blocked; hence, it is also speculated that changes in waveform and SpO_2_ levels may appear more prominently than those in our cuff inflation test. A previous study reported a remote flap monitoring method using a device to send text message alerts to surgeons when the tissue oximetry readings suggested potential flap compromise based on established thresholds [25]. If the threshold can be established by additional research, remote monitoring will be possible even with a pulse oximeter.

## Conclusions

Flap monitoring using a commercially available pulse oximeter and a bedside patient monitor represents an inexpensive, versatile, easy-to-understand, and useful monitoring method. The thigh pressure cuff inflation test revealed that the waveform disappeared during arterial occlusion and that the waveform flattened and SpO_2_ levels decreased slightly during venous occlusion. Further studies are needed to define pulse oximeter changes in cases of blood circulation disorder in clinical flaps.
